# Lipid Peroxidation Is another Potential Mechanism besides Pore-Formation Underlying Hemolysis of Tentacle Extract from the Jellyfish *Cyanea capillata*

**DOI:** 10.3390/md11010067

**Published:** 2013-01-09

**Authors:** Tao Wang, Xiao-Juan Wen, Xiao-Bin Mei, Qian-Qian Wang, Qian He, Jie-Min Zheng, Jie Zhao, Liang Xiao, Li-Ming Zhang

**Affiliations:** 1 Department of Chemical Defense Medicine, Faculty of Naval Medicine, Second Military Medical University, Shanghai 200433, China; E-Mails: wangtao2086@163.com (T.W.); shengnan19871016@163.com (X.-J.W.); abc_w@163.com (Q.-Q.W.); trados1@126.com (J.-M.Z.); zs1010@hotmail.com (J.Z.); 2 Department of Nephrology, Changhai Hospital, Second Military Medical University, Shanghai 200433, China; E-Mail: meixiaobin@medmail.com.cn; 3 Department of Gynecology and Obstetrics, Changhai Hospital, Second Military Medical University, Shanghai 200433, China; E-Mail: heqian1970@yahoo.com.cn

**Keywords:** jellyfish, *Cyanea capillata*, hemolysis, pore-formation, lipid peroxidation

## Abstract

This study was performed to explore other potential mechanisms underlying hemolysis in addition to pore-formation of tentacle extract (TE) from the jellyfish *Cyanea capillata*. A dose-dependent increase of hemolysis was observed in rat erythrocyte suspensions and the hemolytic activity of TE was enhanced in the presence of Ca^2+^, which was attenuated by Ca^2+^ channel blockers (Diltiazem, Verapamil and Nifedipine). Direct intracellular Ca^2+^ increase was observed after TE treatment by confocal laser scanning microscopy, and the Ca^2+^ increase could be depressed by Diltiazem. The osmotic protectant polyethylenglycol (PEG) significantly blocked hemolysis with a molecular mass exceeding 4000 Da. These results support a pore-forming mechanism of TE in the erythrocyte membrane, which is consistent with previous studies by us and other groups. The concentration of malondialdehyde (MDA), an important marker of lipid peroxidation, increased dose-dependently in rat erythrocytes after TE treatment, while *in vitro* hemolysis of TE was inhibited by the antioxidants ascorbic acid—Vitamin C (Vc)—and reduced glutathione (GSH). Furthermore, *in vivo* hemolysis and electrolyte change after TE administration could be partly recovered by Vc. These results indicate that lipid peroxidation is another potential mechanism besides pore-formation underlying the hemolysis of TE, and both Ca^2+^ channel blockers and antioxidants could be useful candidates against the hemolytic activity of jellyfish venoms.

## 1. Introduction

Jellyfish venoms have a wide spectrum of biological activities, such as dermonecrotic, neurotoxic, hemolytic and cardiovascular toxicity [1,2,3]. Among them, hemolytic toxicity is considered to be the most basic damage factor, and nearly all investigated jellyfish venoms possess strong hemolytic toxicity. Hemolysins have been successfully purified and identified from the jellyfish *Carybdea rastoni* [4], *Carybdea alata* [5], *Chiropsalmus quadrigatus* [6], *Chironex fleckeri* [7], and *Cyanea capillata* [8]. According to bioinformatic sequence analysis and investigations of drug intervention against hemolysis [9,10,11], hemolysins may function as a pore-former in cell membrane both *in vivo* and *in vitro* [12,13,14,15]; furthermore, it was found that jellyfish venoms can induce changes of Ca^2+^, K^+^ and Na^+^ flux across the cell membrane, indicating that the pore-former might be a non-selective cation channel complex [12,14]. Moreover, apparent membrane pore-formation was observed in cultured cells by transmission electron microscopy after exposure to jellyﬁsh venoms [13]. Therefore, these pores, formed by aggregates of cytolysin molecules, could lead to cellular lysis through ion and solution imbalance, following the formation of pore-forming complexes by venoms in cell membranes.

Certainly, pore-formation by hemolysins is an important mechanism of hemolysis of jellyfish venoms. However, there might have been enough time (as long as 550–750 million years) for jellyfish to evolve other pathways besides pore-formation to destroy the cell membranes. In view of jellyfish having highly efficient toxic strategies for prey and defense, lipid peroxidation might be another potential mechanism of hemolysis, which can cause loss of polyunsaturated fatty acids, inactivation of membrane enzymes and cytoplasmic proteins, alteration in ion transport and generation of lipid hydroperoxides. It has been attributed as one of the major pathways for explaining the toxicity of many xenobiotics [16,17]. In addition, several sea anemone venoms, for example those from *Actinia equina* and *Bartholomea annulata*, can induce intracellular reactive oxygen species (ROS) formation in cultured cells [18,19]. Some digestive enzymes, acting as toxins, from box jellyﬁsh, sea anemones, and corals induce ROS or lysophospholipid formation thus damaging target cells as small prey or contributing to human envenomation [20]. Therefore, we speculated that oxidative damage might play a role in the hemolytic toxicity or cytotoxicity of TE from the jellyfish *C. capillata*.

In the present study, we first investigated the role of pore-formation in the hemolysis of jellyfish venom with the Ca^2+^ indicator, Ca^2+^ channel blockers and PEG with different molecular weights. We then estimated lipid peroxidation by TE and the effects of antioxidants against the hemolytic activity of TE both *in vitro* and *in vivo*. Accordingly, we proposed that lipid peroxidation may be another potential mechanism underlying hemolysis besides pore-formation by TE from the jellyfish *C. capillata*.

## 2. Results

### 2.1. Hemolytic Activity

**Figure 1:**
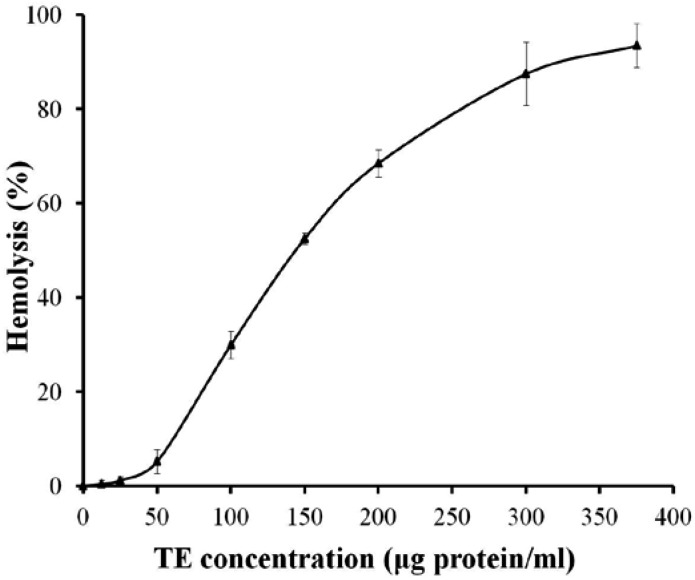
Dose-response curve of hemolytic activity of tentacle extract (TE) from the jellyfish *Cyanea capillata* in rat erythrocyte suspension. All the results are expressed as mean ± SD (*n* = 6).

The hemolytic activity of TE was dose-dependent between 25 and 375 μg protein/mL in 0.45% rat erythrocyte suspension, and the hemolysin unit (HU_50_, is defined as the concentration of protein that causes 50% lysis of erythrocytes) was 149.5 μg protein/mL (Figure 1).

### 2.2. Effects of Ca^2+^ and Ca^2+^ Channel Blockers on the Hemolysis of TE

**Figure 2:**
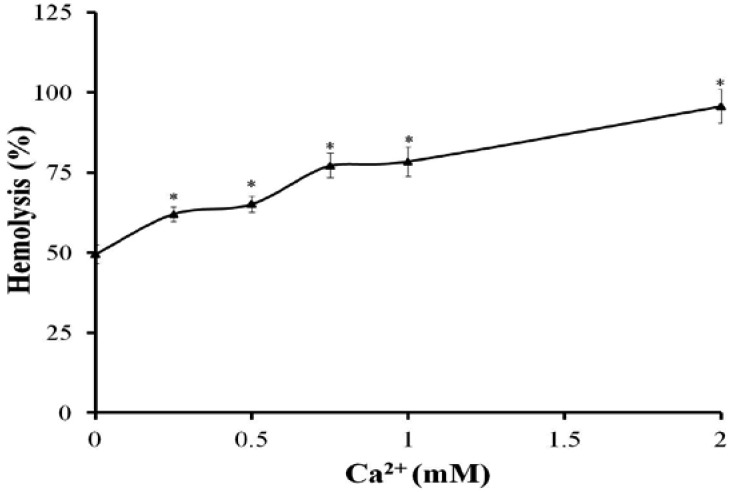
The hemolytic activity of TE (150 μg protein/mL) after treatment with different concentrations of Ca^2+^. The results are expressed as mean ± SD (*n* = 6, * *P* < 0.05 indicated significant differences from TE without Ca^2+^; one-way ANOVA followed by Dunnett’s test).

**Figure 3:**
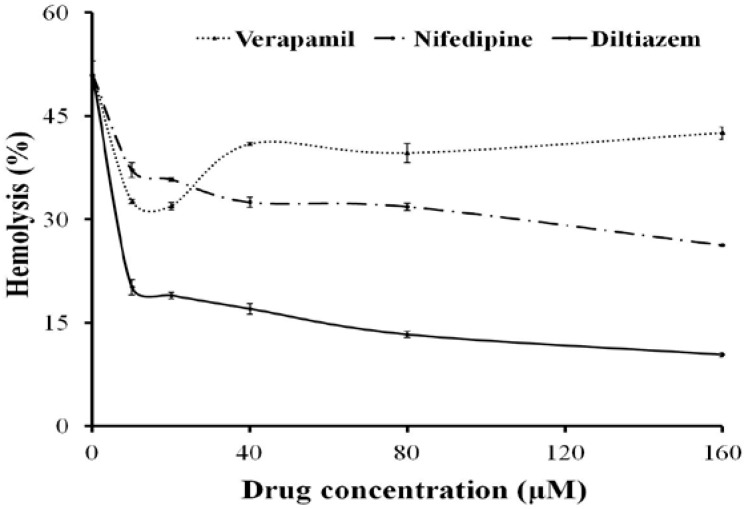
Effects of Ca^2+^ channel blockers on the hemolytic response evoked by TE (150 μg protein/mL). Aliquots of 0.45% erythrocyte suspension pre-incubated with Diltiazem, Nifedipine or Verapamil for 30 min at 37 °C were added into TE and then the hemolytic activity was determined. All the results are expressed as mean ± SD (*n* = 4).

In the presence of Ca^2+^ from 0.25 to 2 mM, the hemolytic activity of TE (150 μg protein/mL) increased signiﬁcantly compared with TE treatment alone without extracellular Ca^2+^ (Figure 2). The Ca^2+^ channel blockers, Diltiazem, Verapamil or Nifedipine, produced a slight hemolysis themselves (<5%), however, all signiﬁcantly inhibited the hemolytic toxicity of TE compared with TE treatment alone, with Diltiazem having the strongest inhibitory effect (79.6% at 160 μM *vs.* TE treatment value) (Figure 3).

### 2.3. Changes of Ca^2+^ in Erythrocytes

**Figure 4:**
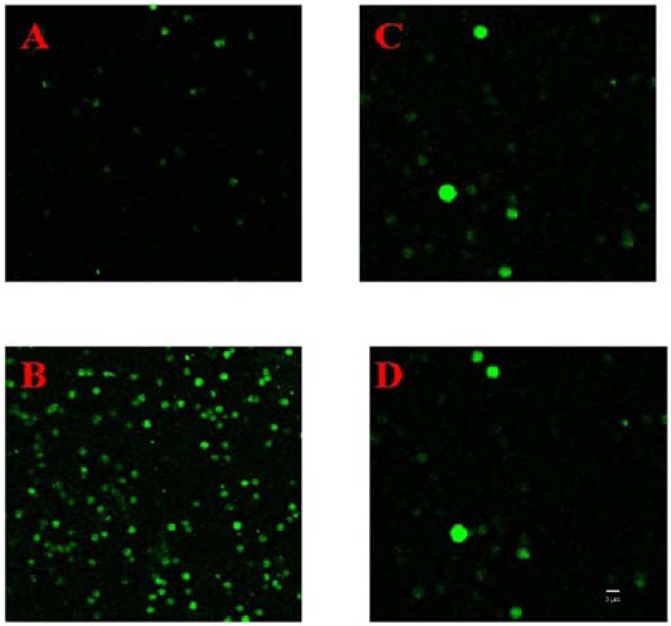
Changes of cytosolic Ca^2+^ fluorescence of red blood cells after exposure to TE (75 μg protein/mL) by confocal laser scanning microscopy: (**A**) Before administration of TE; (**B**) 10 min after administration of TE; (**C**) Pre-incubation with Diltiazem at 37 °C for 30 min but without TE; (**D**) Pre-incubation with Diltiazem for 30 min at 37 °C followed by 10 min after administration of TE (*n* = 3). Bar represents 5 μm.

Ca^2+^ fluorescence in erythrocytes significantly increased from 7.52 ± 1.68 without TE intervention to 164.25 ± 12.87 (*n* = 8) after TE treatment at a concentration of 75 μg protein/mL which would not have led to many erthrocytes lysing rapidly, as shown in Figure 4A,B. When the erythrocytes were pre-incubated with Diltiazem at 37 °C for 30 min, the amplitude of Ca^2+^ fluorescence enhancement by TE treatment was attenuated, (the Ca^2+^ fluorescence increasing from 8.19 ± 2.75 to 13.25 ± 3.19 (*n* = 8), as shown in Figure 4C,D).

### 2.4. Effect of Osmotic Protectants

**Figure 5:**
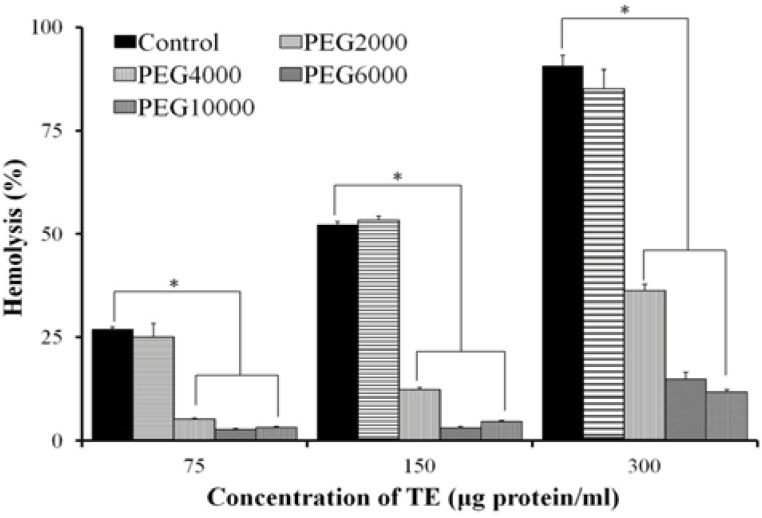
Osmotic protection of hemolytic activity in the treatment series with PEG (PEG2000, PEG4000, PEG6000, PEG10000). TE aliquots (75, 150, 300 μg protein/mL, respectively) were added to a 0.45% erythrocyte suspension containing PEG at a final concentration of 25 mM (*n* = 4, * *P* < 0.05 *vs.* Control; one-way ANOVA followed by Dunnett’s test).

**Figure 6:**
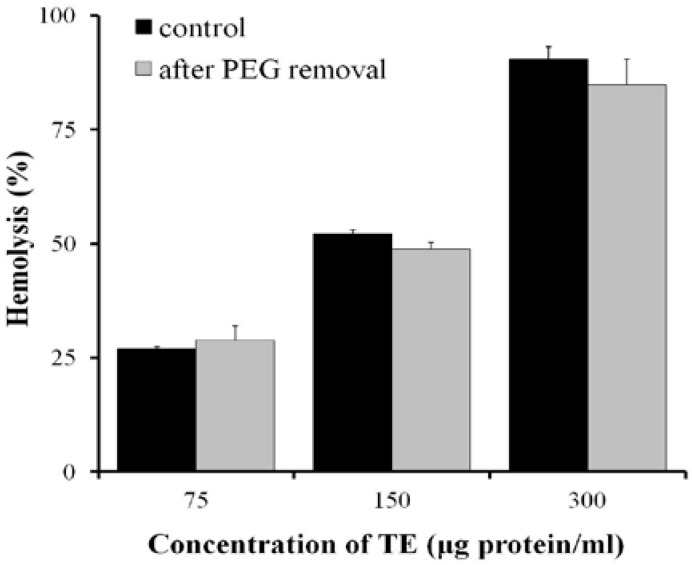
Hemolysis in erythrocytes pre-incubated with 25 mM PEG6000 for 5 min, then PEG washed out twice with phosphate buffered saline (PBS) and different concentrations of TE administered (*n* = 4).

Hemolysis of TE was significantly reduced in the polyethylenglycol (PEG) series with molecular weight (MW) between 4000 Da and 10,000 Da (Figure 5). After pre-incubation with 25 mM PEG6000 for 5 min, the 0.45% erythrocyte suspension was centrifuged at 1000× *g* for 10 min and the supernatant was removed, such a process was repeated two more times to wash out PEG with PBS, the hemolysis of TE was restored to the level without PEG treatment (Figure 6).

### 2.5. Effects of Antioxidants on the Hemolysis of TE *in Vitro*

When incubated with erythrocytes, both Vc (5 mM) and GSH (50 μM) produced a significant attenuation (71.2% and 32.3%, respectively) of the hemolysis of TE at 300 μg protein/mL (*n* = 5, * *P* < 0.05 *vs.* TE treatment alone; one-way ANOVA followed by Dunnett’s test). Vc and GSH did not cause hemolysis themselves in the absence of TE aliquots at the concentration chosen for the experiments.

### 2.6. Lipid Peroxidation

**Figure 7:**
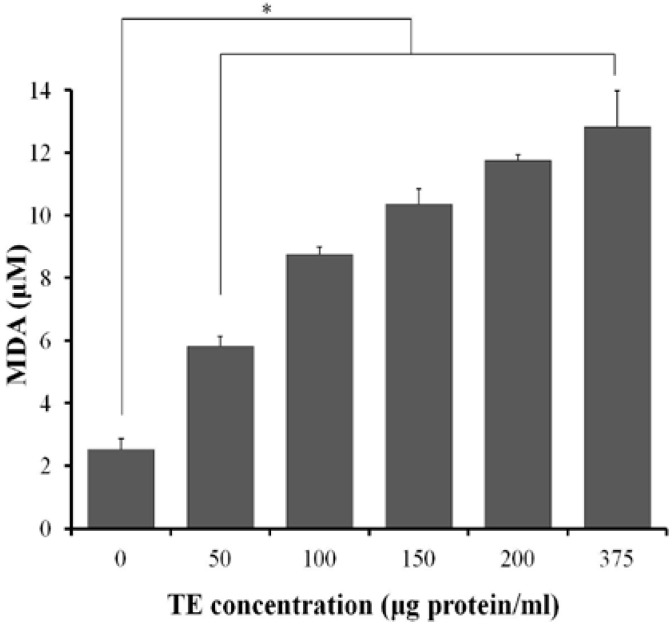
Effect of TE on lipid peroxidation in rat erythrocytes. Results are expressed as mean ± SD (*n* = 4, * *P* < 0.05 indicated significant difference from the control; one-way ANOVA followed by Dunnett’s test).

When exposed to increasing concentrations of TE between 50 and 375 μg protein/mL, rat erythrocytes showed a dose-dependent increase of lipid peroxidation, with the MDA level increasing from 2.52 ± 0.35 μM to 12.81 ± 1.15 μM at the highest dose of TE (Figure 7).

### 2.7. Effect of Vc on the Hemolysis of TE *in Vivo*

**Figure 8:**
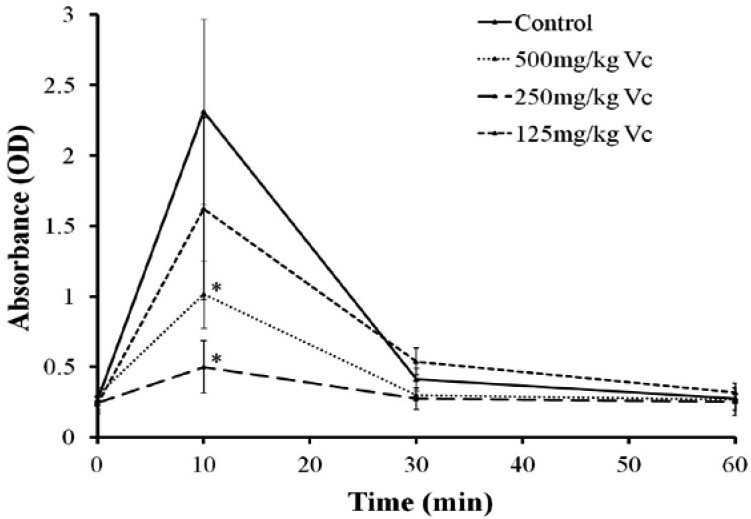
Effect of Vc on the hemolysis of TE (1.25 mg protein/kg i.v.) *in vivo*. The optical density (OD) of rat serum at 415 nm increased and reached a peak at 10 min, while Vitamin C (Vc) (250, 500 mg/kg) reduced the maximum of OD significantly. Results are expressed as mean ± SD (*n* = 8, * *P* < 0.05 indicated significant difference from the control group (intraperitoneal injection of PBS before TE administration) at the corresponding time points; one-way ANOVA followed by Dunnett’s test).

After TE administration (1.25 mg protein/kg), the optical density (OD) of rat serum at 415 nm increased sharply and reached a peak after 10 min, then gradually returned to the baseline level in the following 60 min, while Vc (250, 500 mg/kg) treatment evidently attenuated the peak of OD (Figure 8). The electrolytes Na^+^, K^+^, Ca^2+^, Cl^−^ and lactic acid (Lac) were all significantly influenced by TE with Na^+^, Ca^2+ ^and Cl^−^ decreasing and K^+ ^and Lac increasing 10 min after TE administration. The amplitude of K^+^ and Lac increase was also significantly attenuated by Vc (250 mg/kg) (Table 1).

**Table 1:** Effects of Vitamin C (Vc) on the electrolyte change induced by tentacle extract (TE) *in vivo* (*n* = 6, Δ *P* < 0.05 *vs.* control group (pretreated with phosphate buffered saline (PBS) before PBS administration), ▲ *P* < 0.05 *vs.* TE group; one-way ANOVA).

	Na^+^ (mM)	K^+^ (mM)	Ca^2+^ (mM)	Cl^−^ (mM)	Lac (mM)
Control	139.8 ± 1.33	7.23 ± 0.44	2.34 ± 0.08	99.8 ± 1.70	3.18 ± 0.40
TE (1.25 mg/kg)	137.5 ± 1.05 Δ	10.65 ± 1.31 Δ	2.20 ± 0.05 Δ	94.7 ± 0.82 Δ	8.02 ± 0.43 Δ
Vc (250 mg/kg) + TE (1.25 mg/kg)	136.0 ± 1.10 Δ	8.42 ± 0.72 Δ▲	2.26 ± 0.04 Δ	93.7 ± 0.52 Δ	4.98 ± 0.85 Δ▲

## 3. Discussion

It is believed that the toxic effects of jellyfish venoms are caused by the combined action of various toxic components [2,3,21]. Hemolysin is one of the important damage factors, contributing to the *in vivo* toxic effects of jellyfish venoms through direct hemolysis [13,14,15] or through indirect damage by elevating blood K^+^ concentration and serious acidosis, which can associate with other toxicity such as cardiovascular and muscular toxicity [22,23,24]. Using the TE from the jellyfish *C. capillata*, we tried to explore the exact mechanism underlying the hemolysis of jellyfish venom, in order to deepen the understanding of the hemolysis process and finally prompt new research on the prevention and the therapy strategy against jellyfish stings*.*

Our laboratory conducted a series of studies on the hemolytic toxicity of TE from the jellyfish *C. capillata*. We confirmed that both the extract of the tentacle tissue and the nematocyst venom possess strong hemolytic toxicity and we further investigated the physical and chemical conditions influencing the hemolytic toxicity of jellyfish venom [25]. Our previous experiments showed that non-specific cation channel blocker La^3+^ can significantly inhibit the hemolytic toxicity of TE [26]. Considering the scarcity of ion channels in the erythrocyte membrane, it seems unlikely that TE plays a role by stimulating the membrane ion channels. Therefore, these results suggested that TE from the jellyfish *C. capillata* may execute a hemolytic effect through transmembrane pore formation in erythrocyte membranes [8,22].

In the present study, we first examined the hemolytic toxicity of TE, and further confirmed the enhancement of hemolytic toxicity by extracellular Ca^2+^ within 2 mM, which was consistent with our previous results and those from other research groups [26,27]. With Ca^2+^ as the indicator of transmembrane pore formation, we found that intracellular Ca^2+^ concentration increased significantly in rat erythrocytes after TE administration. Considering the lack of Ca^2+^ storage organelles such as endoplasmic reticulum and mitochondria in erythrocytes, as well as the scarcity of Ca^2+^ channel in the erythrocyte membrane, the increase of intracellular Ca^2+^ concentration must be due to the influx of extracellular Ca^2+^ through the channels formed by TE. The increase of serum K^+^ and decrease of serum Ca^2+^ and Na^+^ further supported the fact that the channel formed by TE is a non-specific cation channel complex. 

In addition, osmotic protection assay showed that the hemolysis of TE was impaired by PEG with MW between 4000 Da and 10,000 Da. When the erythrocyte suspension was pre-incubated with PEG6000 for 5 min and then the PEG6000 was washed out, the hemolysis of TE was restored to the level without PEG treatment, suggesting membrane lesion by TE with diameters comparable to those of osmotic protectants and thus enhancing the hypothesis of the pore-forming mechanism underlying the hemolysis of TE. Interestingly, the L-Ca^2+^ channel blockers, Diltiazem, Verapamil and Nifedipine, significantly inhibited the hemolytic activity of TE, and furthermore, Diltiazem attenuated the increase of intracellular Ca^2+^ concentration. These results are fairly significant for the prevention and therapy of jellyfish stings, since several researchers have reported that Ca^2+^ channel blockers can inhibit the cardiovascular toxicity of jellyfish venoms as well [28,29].

Cytolytic toxins are known to operate by ways either enzymatic, producing a direct lytic action upon erythrocytes, or stoichiometric, with the stoichiometric lysins including pore formers and lipid peroxidation [30,31]. Lipid peroxidation is a common mechanism for cell injury. Vertebrate red blood cell membranes are common targets for oxidative damage by peroxidation because they have a high content of unsaturated lipids as well as iron in hemoglobin, one of the most powerful catalysts capable of initiating lipid peroxidation. In addition, erythrocyte membrane proteins are susceptible to covalent damage, including cross-linking and aggregation, by oxygen radical-induced lipid peroxidation products. The lipid peroxidation can impair membrane function, decrease ﬂuidity, inactivate membrane-bound receptors and enzymes, and increase non-speciﬁc permeability to ions such as Ca^2+^ [17,32]. The increase in intracellular Ca^2+^ concentration could further activate phospholipases and proteases, leading to free radical reactions involving membrane damage. Our present studies have determined a significant dose-dependent increase of the oxidative stress biomarker MDA, as well as the inhibition of antioxidant against the hemolysis by TE both *in vitro* and *in vivo*, indicating that the lipid peroxidation might be another important hemolysis mechanism of TE from the jellyfish *C. capillata*.

It has been reported that the stimulation of lipid peroxidation as a consequence of iron ions released from erythrocytes may increase the permeability to ions such as Ca^2+^, suggesting the peroxidative process is related to the increase in cytosolic Ca^2+^ [33]. Lipid peroxidation can also produce “holes” in erythrocyte membranes of roughly 70 Å by disrupting membrane phospholipids involved in maintaining the integrity of the plasma membrane [34]. Whether pore-formation is mainly attributed to lipid peroxidation or to self-assembly of hemolysin could be an important question underlying the mechanism of TE hemolysis. Purifying the oxidative component and going into its structural details according to the structure-function relationship might be a possible way to further prove the oxidative effect of TE. So far, the hypothesis that TE forms pores in the cell membrane through oxidative damage of plasma membrane certainly needs more experimental evidence and this will be our next main research issue.

## 4. Experimental Section

### 4.1. TE Preparation from the Jellyfish *C. capillata*

Specimens of *C. capillata* were collected in June 2010 in the Sanmenwan offing of the East China Sea in Zhejiang Province, China, and identified by Professor Huixin Hong from the Fisheries College of Jimei University, Xiamen, China. The removed tentacles were preserved in plastic bags on dry ice and immediately shipped to Shanghai, where the samples were frozen at −70 °C until use. The TE was prepared following the method described as in previous reports [25,35]. Briefly, frozen tentacles were thawed at 4 °C in filtered seawater at a mass/volume ratio of 1:1 to allow autolysis of the tissues for 4 days. The mixture was then stirred for 30 min twice daily. The autolyzed mixture was centrifuged at 10,000× *g* for 15 min three times. The resultant supernatant was the TE. All procedures were performed at 4 °C or in an ice bath. Before being used for injection, TE was centrifuged at 10,000× *g* for 15 min to remove sediment, followed by dialysis against PBS (0.01 M, pH 7.4) at 4 °C for over 8 h. Protein concentrations were determined in all samples by the method of Bradford (1976), with fetal bovine serum albumin (high purity, Sigma, St. Louis, MO, USA) as a standard [36].

### 4.2. Hemolytic Activity Assay

Hemolytic activity of TE was tested in 0.45% male rat erythrocyte suspension. In brief, 100 μL of fresh heparinized blood samples were made up to 10 mL in phosphate buffered saline (PBS, self prepared, with the following composition in mM: 135 NaCl, 4.7 KCl, 10 Na_2_HPO_4_, 2 NaH_2_PO_4_, pH 7.4), and then centrifuged at 1000× *g* for 10 min at 4 °C. The supernatant and buffy coat were removed by gentle aspiration, and the above process was repeated two more times. Erythrocytes were finally resuspended in the same buffer to a ﬁnal concentration of 0.45% (v/v). Aliquots of 0.45% erythrocyte suspension were incubated at 37 °C for 30 min in the presence of TE with the concentrations ranging from 0 to 375 μg protein/mL (50% v/v), and then centrifuged at 1000× *g* for 10 min at 4 °C to precipitate both the intact erythrocytes and ghosts. The 200 μL supernatants were transferred into 96-well microplates and the absorbance at 415 nm was determined by using a spectrophotometric microplate reader (BIO-RAD iMark™, Hercules, CA, USA) to measure the extent of red blood cell lysis. The hemolytic activity of TE was expressed as % absorbance compared with that observed after maximal lysis induced by sodium dodecyl sulphate (40 μg/mL). The supernatant of untreated 0.45% erythrocyte suspension was taken as the background and subtracted (*n* = 6). Male Sprague-Dawley (SD) rats (220–240 g) were provided by the Laboratory Animal Center of the Second Military Medical University, Shanghai. All were procured from the animal care facility at the university where they were housed in cages with 12/12 h light/dark cycle at 22 ± 2 °C and given standard diets plus water *ad libitum*. The investigation was carried out in conformity with the Ethics Committee of the Second Military Medical University and international guidelines for animal handling.

### 4.3. Effects of Ca^2+^ and Ca^2+^ Channel Blockers on the Hemolysis of TE

The effect of Ca^2+^ was studied by adding CaCl_2_ into TE and then the hemolytic activity was assayed as described above. Ca^2+^ was employed at a final concentration of 0.25, 0.5, 0.75, 1.0 and 2.0 mM, respectively. To inspect the possible protective effect of Ca^2+^ channel blockers against hemolysis, Diltiazem, Verapamil and Nifedipine (Sinopharm Chemical Reagent Co. Ltd., Shanghai, China) were incubated with 0.45% erythrocyte suspension at 37 °C for 30 min before TE administration (150 μg protein/mL). Then the relative percentage of hemolytic activity was calculated as described above. All the Ca^2+^ channel blockers were employed at final concentrations of 10, 20, 40, 80 and 160 μM, respectively.

### 4.4. Measurement of Cytosolic Free Ca^2+^ Using CLSM

The intracellular Ca^2+^ indicator fluo-3 acetoxymethyl (AM) was used to measure the Ca^2+^ changes in the erythrocyte. Briefly, 1 mL of the 0.2% erythrocyte suspension was loaded with Fluo-3/AM (5 μM) and further incubated at 37 °C for 120 min in the dark. After this, the solution was centrifuged at 1000× *g* for 10 min and the supernatant was removed. Such a process was repeated two more times. The erythrocytes were finally resuspended in 1 mL Hank’s solution (Sangon Biotechnology, Shanghai, China, with the following composition in mM: 1.6 CaCl_2_, 1.0 MgCl_2_, 145 NaCl, 5.0 KCl, 0.5 NaH_2_PO_4_, 10 dextrose, and 10 Hepes, pH 7.4). To test the influence of Ca^2+^ channel blocker on the changes of intracellular Ca^2+^ induced by the TE, 0.2% erythrocyte suspension was pre-incubated with Diltiazem (100 μM) before exposure to the TE (75 μg protein/mL). For confocal laser scanning microscopy, 200 μL of such a solution with or without Diltiazem was replated onto special dishes. Intracellular Ca^2+^ changes were measured by monitoring the alteration of cell fluorescence.

### 4.5. Osmotic Protection Assay

To test the effect of osmotic protectants, 25 mM PEG with different molecular weights (2000, 4000, 6000 and 10,000 Da) was added to 1 mL 0.45% erythrocyte suspension. Then aliquots of TE (75, 150, 300 μg protein/mL) were added, and the hemolytic activity was detected as described above. In another separate experiment, 25 mM PEG6000 was pre-incubated with 0.45% erythrocyte suspension at 37 °C for 5 min, then the 0.45% erythrocyte was centrifuged at 1000× *g* for 10 min and the supernatant was removed. Such a process was repeated two more times to wash out PEG with PBS, and the erythrocytes were finally resuspended in PBS to a final concentration of 0.45% (v/v), and then aliquots of TE (75, 150, 300 μg protein/mL) were added to test the hemolytic activity. 

### 4.6. Effects of Antioxidants on the Hemolysis of TE *in Vitro*

In order to test the possible involvement of lipid peroxidation in the hemolysis, aliquots of 0.45% erythrocyte suspension were pre-treated with Vc and GSH at 37 °C for 30 min at a ﬁnal concentration of 5 mM and 50 μM, respectively, before TE (300 μg protein/mL) administration, and hemolytic activity was detected as described in Section 4.2.

### 4.7. Measurement of Malondialdehyde

For lipid peroxidation assay, we used a commercial kit (Beyotime Institute of Biotechnology, Nantong, Jiangsu, China) to quantify the generation of malondialdehyde (MDA) according to the manufacturer’s protocol. In brief, the 20% erythrocyte suspension aliquots were incubated with TE at 37 °C for 30 min and then centrifuged at 1000× *g* for 10 min, and the supernatant was subjected to measurement of MDA levels. 

### 4.8. Effect of Vc on the Hemolysis of TE *in Vivo*

Thirty-two male Sprague-Dawley (SD) rats (220–240 g, provided by the Laboratory Animal Center of the Second Military Medical University, Shanghai, China) were randomized into four groups (*n* = 8) and anesthetized with urethane (1.2 g/kg i.p.). To inspect the possible protective effect of antioxidants against hemolysis of the TE *in vivo*, Vc at different doses (125, 250, 500 mg/kg i.p.) was adopted before TE administration (1.25 mg protein/kg i.v.). The blood sample was obtained through a glass capillary inserted into the retrobulbar venous plexus at 0 (pre-injection), 10, 30 and 60 min after TE administration, respectively. Similarly, the rats of the control group (*n* = 8), after intraperitoneal injection of PBS, were treated with TE through tail vein injection and blood samples were obtained at the corresponding time points. Aliquots of the blood samples were centrifuged at 1000× *g* for 10 min to precipitate both the intact erythrocytes and ghosts. The supernatants were collected as the serum and the ODs were spectrophotometrically measured at 415 nm as the marker of hemolytic activity *in vivo*.

To further observe the effects of antioxidants on the changes of electrolytes and Lac *in vivo*, eighteen male SD rats were randomized into three groups (*n* = 6) and pretreated with PBS or Vc (250 mg/kg i.p.), then TE at the dose of 1.25 mg protein/kg was chosen for tail vein injection and the serum was collected 10 min after TE administration. The sham-treated animals (*n* = 6) were administered with the same volume of PBS. Electrolytes (Na^+^, K^+^, Ca^2+^ and Cl^−^) and Lac in the serum collected as described above were analyzed by an arterial blood gas analyzer (GEM Premier 3000, International Laboratory, Bedford, MA, USA).

### 4.9. Statistical Analysis

One-way analysis of variance (ANOVA) was used. In all cases, statistical significance was indicated by *P* < 0.05. All data were expressed as mean ± SD.

## 5. Conclusions

We further elucidated that non-specific cation transmembrane pore formation is one of the mechanisms underlying hemolysis of TE from the jellyfish *C. capillata*. Furthermore, we proposed that lipid peroxidation might be another potential mechanism of hemolysis, in addition to pore-formation. Both Ca^2+^ channel blockers and antioxidants might be considered as useful candidates against the hemolytic activity of jellyfish venoms. 
